# Primary Melanoma of the Pineal Gland Case Report and Review of the Literature

**DOI:** 10.3390/reports7020049

**Published:** 2024-06-20

**Authors:** Daniel Rotariu, Bogdan F. Iliescu, Gabriela Dumitrescu, Antonia Nita, Bogdan Costachescu

**Affiliations:** 1 Department of Neurosurgery, “Gr T Popa” University of Medicine and Pharmacy Iasi, 11 Universitatii St., 700115 Iasi, Romania; Daniel-ilie.rotariu@umfiasi.ro (D.R.); bogdan.costachescu@umfiasi.ro (B.C.); 2 “Prof Dr N Oblu” Clinical Emergency Hospital, 700309 Iasi, Romania; dr_gabriela_dumitrescu1965@yahoo.com (G.D.); nita_antonia@yahoo.com (A.N.)

**Keywords:** pineal, melanoma, leptomeningeal metastasis, endoscopy

## Abstract

Pineal-region tumors are a histologically heterogeneous group of tumors and represent a rare occurrence, accounting for less than 1% of all adult intracranial tumors. Among these, primary pineal malignant melanomas (PPM) represent an even rarer entity, with only twenty-five cases being reported in the literature to date. We present the case of a 65-year-old patient who presented in our department for progressive headache, gait disturbance and memory impairment. Magnetic resonance imaging (MRI) of the brain revealed a solid mass in pineal region, measuring 2.2 × 1.2 × 2.0 cm and causing obstructive hydrocephalus. He underwent a third ventriculostomy, but we failed to obtain a sample for diagnostic purposes. The intraoperative surprise was the presence, at the level of the third ventricle, of multiple melanin deposits, which were not picked up by the MRI. Although the biopsy could not be performed and had to be obtained by stereotactic biopsy in a second intervention, the endoscopy findings allowed for the correct staging of the intracranial disease and appropriate treatment management.

## 1. Introduction

Pineal region tumors are a histologically heterogeneous group of tumors and represent a rare occurrence, accounting for less than 1% of all adult intracranial tumors. Among these, primary pineal malignant melanomas (PPM) represent an even rarer entity, with only twenty-five cases being reported in the literature to date.

PPMs are likely to arise from cells within the leptomeninges surrounding the pineal gland that will further invade and replace the gland [1]. The outcome for patients with a PPM is poor, particularly for the cases with associated leptomeningeal dissemination [1,2].

We present the first case of a PMM with intraoperative endoscopic diagnosis of leptomeningeal seeding before the imaging could depict it.

## 2. Detailed Case Description

The patient was a 65-year-old man with no significant medical history who presented in our department with a 3-month history of progressive headache, gait disturbance and memory impairment.

On neurological examination, the patient was alert and oriented. He complained of eyestrain headaches, worsening gait imbalance with difficulties performing tasks requiring fine coordination and trouble remembering recent events.

Hematologic findings, including a complete blood count, electrolyte levels, erythrocyte sedimentation rate, hepatic enzyme levels, including serum-alfa fetoprotein, human chorionic gonadotrophin, fluid placental alkaline phosphatase and lactic dehydrogenase assays, were all normal.

**Figure 1:**
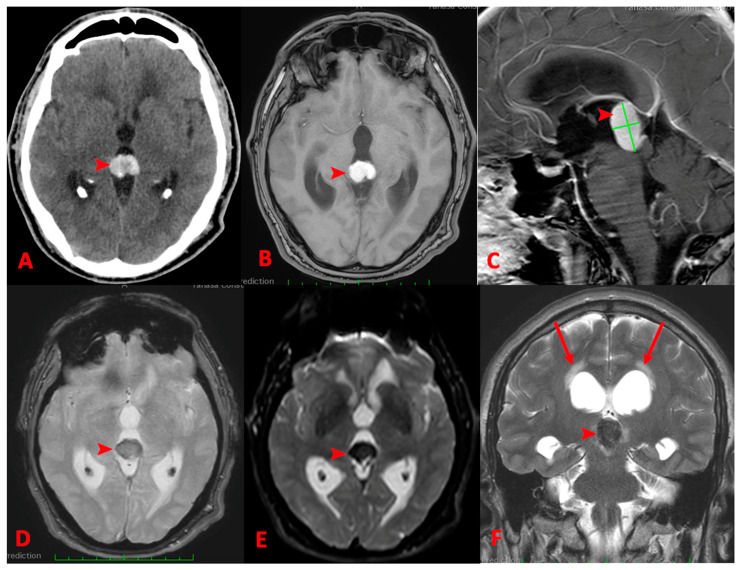
Preoperative images (**A**) nonenhanced computer tomography showing hyperdense mass at the level of pineal gland. The lesions are hyperintense in T1 (**B**,**C**), hypointense in T2 (**D**), and exhibit marked reduced diffusion on DWI (**E**). The lesion is compressing the tectal plate, obstructing the cerebral aqueduct and determining active hydrocephalus (arrows) (**F**).

Computed tomography showed a hyperdense mass at the level of the pineal gland and magnetic resonance imaging (MRI) of the brain revealed a solid mass in the pineal region, causing obstructive hydrocephalus. (Figure 1A–F). The tumor measured 2.2 × 1.2 × 2.0 cm.

The patient was scheduled for endoscopic third ventriculostomy and biopsy of the pineal mass.

The surgical intervention was performed under general anesthesia, with the patient in a prone position and the head fixed in a Mayfield head holder for neuronavigation (StealthStation 8 Medtronic^®^). This was used as an adjunct for guidance.

**Figure 2:**
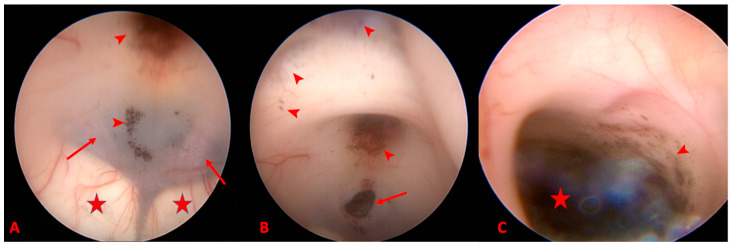
Intraoperative images, (**A**) anterior portion of the 3rd ventricle floor showing the mammillary bodies (star), and tuber cinereum through which posterior cerebral arteries (arrows) can be observed, along with tumoral dissemination at the level of the pituitary infundibulum and tuber cinereum (arrow heads). (**B**) Intraoperative aspect showing the stoma (arrow) and other disseminations at the level of the optic chiasm and lamina terminalis (arrow heads). (**C**) Endoscopic view of the posterior 3rd ventricle showing the black lesion and the involvement of the tegmentum.

A right frontal burr hole was used for the endoscopic approach. Once the endoscope within the ventricular system was advanced at the level of the third ventricle, where anatomic landmarks were identified, ETV was performed uneventfully using the ETV technique described in previous reports [3]. Notably, at the level of the third ventricle, multiple melanin deposits were identified (Figure 2). These were diffusely spread with the involvement of the tuber cinereum, infundibulum, optic chiasm, lamina terminalis and posterior floor of the third ventricle. CSF samples were taken for cytopathologic examination, which failed to identify melanocytic cells.

After ETV was performed, the endoscope was oriented posteriorly. The lesion was identified, but due to the working angle, a biopsy was not possible. It is known that for lesions located posteriorly to the adhesion intertalamico, a more anterior trajectory is necessary for biopsy [4], and for this reason, a second burr hole was placed more anteriorly using a linear skin incision in a crease in the forehead. Unfortunately, canulation of the ventricle was not possible due to the brain shift that emerged after the loss of CSF during the ETV.

The postoperative course was uneventful, and the patient was discharged on day 2, with resolution of headaches and improvement of gait.

The patient was scheduled for stereotactic biopsy within 1 week. The surgical intervention was performed under general anesthesia, using the Stealth Station 8 biopsy kit from Medtronic^®^, using a right frontal trajectory. The biopsy revealed black/brown and friable material, with a frozen section showing melanotic tissue.

After the final histopathologic exam, the patient was evaluated for a potential primary lesion outside the CNS. He underwent dermatologic evaluation through digital dermoscopy using the FotoFinder^®^ ATBM master, which revealed no evidence of any other primary skin lesion.

Thorough thoracic, abdominal and pelvic CT scan evaluations failed to demonstrate any other lesions. Ophthalmologic examination excluded any form of uveal melanoma.

**Figure 3:**
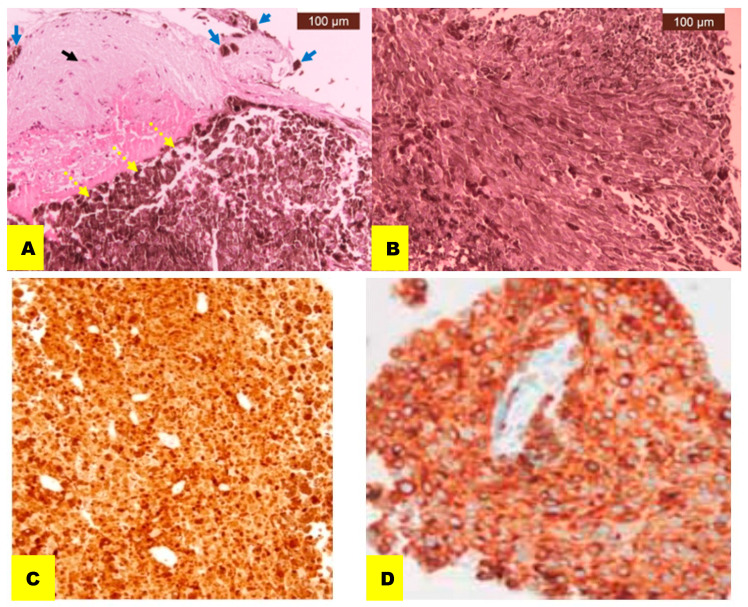
Pathological examination. (**A**) Nervous tissue with a relatively normal appearance (black arrow). Its subpial side is infiltrated by small groups of tumor cells loaded cytoplasmically with a brown granular pigment (melanin) (blue arrows), and on the opposite side its structure is replaced by a densely cellularized tumor, made up of atypical cell sheets with cytoplasm loaded with abundant granular brown pigment resembling melanin (yellow arrows) (HE, ×20). (**B**) Sheets of voluminous tumor cells, with a fusiform appearance, arranged in thick fascicles that intertwine in different directions. Tumor cells show nuclear pleomorphism and an abundant granular brown pigment resembling melanin in their cytoplasm (HE, ×200). (**C**) S100 staining, ×200. (**D**) Mart1 staining, ×200.

The immunohistochemistry exam stained positive for S100 and MART1, and the Ki67 index was positive in 5% of the cells. Molecular diagnosis was not available at the time of diagnosis (Figure 3).

Given the HP result, and the lack of identification of skin or uveal lesion, the final diagnosis was primary melanoma of the pineal gland.

The endoscopic appearance at the first surgery, showing subpial lesion at the level of the ventricular ependyma, prevented a surgical intervention for the resection of the pineal lesion. The tumor was considered as disseminated at the level of the ventricular system and the patient was recommended to undergo radiotherapy and chemotherapy.

The patient underwent stereotactic irradiation, DT 30Gy, in five fractions within 1 month after the final diagnostic.

The 4-month control MRI showed minimal enlargement of the lesion that, at the time, was interpretated as pseudo progression. Treatment with temozolomide was initiated; however, the tumor continued to progress, as was demonstrated by the 8-month control MRI.

**Figure 4:**
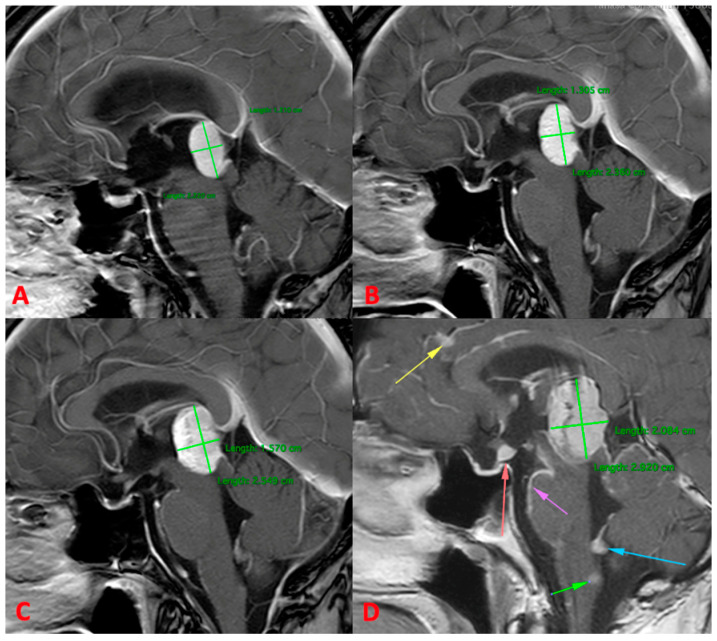
MRI images showing the evolution of the lesion from the diagnosis (**A**) and after radiation therapy at 4 months (**B**) and 12 months (**C**), showing moderate progression and (**D**) MRI at 16 months, showing important growth of the lesion and leptomeningeal spreading with multiple intracranial metastases (arrows).

The patient had a good quality of life for 12 months, despite the slow imagistic progression of the primary lesion, but after that, at 16 months, the patient showed rapid clinical and imagistic progression. Clinically, he had a sixth nerve palsy, Parinaud syndrome, diabetis insipidus and gait disturbances, and on the MRI, notable growth of the lesion and leptomeningeal spreading with multiple intracranial metastases (Figure 4D) were observed. At this stage, the patient was recommended to begin palliative treatment.

## 3. Discussion

The occurrence of a primary melanoma in the pineal gland is very rare, with only 28 cases being reported in the English literature to date (please refer to Table 1 for the choice of treatment in each reported case).

### 3.1. Origin

Primary intracranial melanomas are thought to develop from melanocytes originating from the neural crest during embryogenesis and actively migrating to peripheral sites, such as the skin, mucous membranes, leptomeninges, and uvea [20]. Currently, there are two theories: the first is that primary melanomas arise from melanocytes that are present in the arachnoid surrounding the pineal gland [13,26], and the second one is that primary pineal melanomas are arising from the pineal gland, which has a cell arrangement similar to that of the developing retina, with abundant melanin present in the perinatal period. Although these pigmented cells are supposed to disappear in later development, some may remain and later transform as a result of primary pineal melanoma [17].

### 3.2. Diagnosis

#### 3.2.1. Presentation

Frequently, pineal melanomas present with signs and symptoms of obstructive hydrocephalus, a potentially fatal condition that has to be addressed in an emergency setting. Other symptoms are given by compression of the adjacent structures: tectal plate with upward gaze paralysis and cerebellar ataxia from midbrain compression [19].

#### 3.2.2. MRI

Melanocytic neoplasms appear iso- to hyperdense with homogenous contrast enhancement on a CT scan of the brain. The melanotic type contains more than 10% of melanin-containing cells. The MRI image characteristics of the melanocytic tumors is given by the paramagnetic property of melanin, which is responsible for shortened T1 relaxation time, rendering this group hyperintense on T1-weighted images, hypointense on T2-weighted images, and isointense or hyperintense on proton density-weighted MR images. The amelanotic type comprises less than 10% melanin-containing cells and a particular appearance on an MRI, and it is hypointense on T1- and hyperintense on T2-weighted MR images [2,27]. Differential diagnosis should be made with other pineal masses, as shown in Table 2 [31], but given the heterogenous MRI appearance [2], pure imagistic diagnosis may be difficult.

Definitive diagnosis is made based on histologic appearance (the presence of intracellular melanin), immunohistochemical profile and ultrastructural features. Primary pineal malignant melanoma shows large, pigmented tumor cells that are growing in loose nests, and demonstrates various degrees of pigmentation, from dense to amelanotic, highly variable cytological atypia and mitotic activity, necrosis, increased Ki-67 index >3% and invasion of the surrounding CNS tissue. Immunohistochemical stains are performed on HBM-45, S-100 and melanin A (MART1) (after bleaching) [2].

To distinguish between primary CNS and metastatic melanoma and make the definitive diagnosis, it is important to obtain histologic confirmation of melanocytic origin [27] and to perform extensive investigations, including whole-body FDG PET studies. This is the cornerstone for staging secondary lesions from melanoma, and scrupulous examination by dermatologists has to be undertaken [26,32].

#### 3.2.3. Two

The molecular pathologic diagnosis was proposed by Cornejo et al., who observed that primary lesions in the CNS often have mutations in guanine nucleotide-binding protein (GNAQ) and GNAQ subunit α-11 (GNA11). These mutations are present in 40% of uveal melanomas but are rare in cutaneous and mucosal melanomas [33].

In order to obtain sample tissue, two main techniques are used: stereotactic biopsy and the endoscopic biopsy, with overall similar success rates.

The endoscopic procedures also allow simultaneous treatment of hydrocephalus, and inspection of the ventricular system in addition to tissue sampling [34]. Unfortunately, sometimes, two burr holes may be needed (lesions behind the adhesion interthalamico) [4]. These situations may pose a particular challenge in canulating the ventricle through the second burr due to brain shift after ETV. An alternative for that would be the use of a single burr with the use of a combination of flexible and rigid endoscopes [35]. The stereotactic technique, on the other hand, offers bigger tissue samples compared to the endoscopic technique, with higher diagnostic accuracy (81.1% in endoscopic biopsy vs. 93.7% in stereotactic biopsy) [36].

### 3.3. Treatment

There are no known guidelines for the treatment of primary pineal melanoma. Previous reports suggest that this patient population may be best served by a combination of gross total resection (GTR) followed by RT and systemic therapy [13,17]. Aggressive surgical resection may offer long-term medical and radiologic control and even may influence the life expectancy [27], but unfortunately, GTR is often technically challenging in this region given anatomic relations with the vascular structures, internal cerebral veins and the vein of Galen and the nervous structures, midbrain and thalamus [28].

Radiotherapy is the cornerstone treatment for pineal melanoma, as adjunct of surgery or as standalone treatment, with data in the literature suggesting that it may be an efficient surgical option for long-term control of primary CNS melanoma [37]. Gamma-knife radiosurgery may be an option for residual tumor after partial resection or in recurrent lesions without meningeal spread [27,38].

The role of chemotherapy in primary CNS melanoma is unclear. The most common molecule used is temozolomide, due to the penetration of the blood–brain barrier and is used as monotherapy [24,27]. Immunotherapy (Vemurafenib) is used for the treatment of metastatic melanoma, but its diffusion through the blood–brain barrier remains uncertain [27].

The treatment decision, in our case, was made according to the intraoperative findings of the ETV procedure (Figure 2). Despite the good clinical condition, the patient’s age and the absence of other lesions in the body, we decided not to perform surgical resection based on the aspect of the ventricular ependyma, as multiple pigmentated lesions spread at the level of the ventricles that were considered as disseminated melanoma. From there, we made the decision to perform radiotherapy, with adjuvant treatment with temozolomide.

### 3.4. Prognosis

One of the factors that should be emphasized is that meningeal dissemination is one of the conditioning factors of survival. Longer survival intervals (>4 years) are cited in the literature only with tumor biopsy and chemotherapy, in the absence of meningeal spread [14].

Leptomeningeal spread appears to be common in primary pineal melanoma, and endoscopy may identify it early in the course of the disease, long before it becomes evident on MRI images. This was true in our case, where during the ETV procedure a dark material (melanin) was seen as a diffuse lining in the third ventricle Figure 2; similar findings have been documented by Aaroe et al. [30].

Systemic treatment will play an important role in the treatment of disseminated disease, as will the development of novel carriers for cytotoxic agents at the level of the CNS, such as Cryptotanshinone [39,40] and Juglone [41], which are able to induce apoptosis and kill tumor cells without apparent toxicity to the normal cells.

## 4. Conclusions

We present the first case of primary pineal melanoma with leptomeningeal metastatic lesions that were initially documented during the endoscopic intervention, despite the fact that they were not discernable on an MRI. This finding changed the clinical staging of the disease and the therapeutic protocol according to existing best practices. This finding pleads for an operative attempt to obtain biopsy material from pineal region tumors, as it allows the visualization of ventricular system and can document the real extent of the disease.

## Figures and Tables

**Table 1:** Choice of treatment in all the cases reported in the literature to date.

Case No	Year	Age	Sex	Treatment
1	1899 [5]	32	F	N/A
2	1904 [6]	31	M	N/A
3	1931 [7]	49	M	N/A
4	1957 [8]	68	F	N/A
5	1973 [9]	43	M	N/A
6	1977 [10]	56	M	Radiotherapy
7	1987 [11]	77	F	Biopsy, VPS
8	1988 [12]	59	M	Biopsy
9	1993 [13]	60	M	Resection, Radiotherapy
10	1994 [14]	53	F	Resection, Chemotherapy
11	1998 [15]	49	M	Biopsy
12	2000 [16]	48	M	N/A
13	2001 [17]	50	F	Resection, Radiotherapy
14	2007 [18]	20	F	Biopsy, Radiotherapy, Chemotherapy, VPS
15	2007 [19]	73	F	Radiotherapy
16	2009 [20]	44	M	Radiotherapy, Chemotherapy
17	2011 [21]	70	M	Resection, Radiotherapy
18	2011 [2]	54	F	Resection, Biopsy, Radiotherapy, Chemotherapy, VPS
19	2012 [22]	49	F	Resection, Radiotherapy
20	2012 [1]	22	F	Resection, Biopsy
21	2014 [23]	59	M	Resection, Radiotherapy
22	2015 [24]	45	F	Resection, Radiotherapy
23	2015 [25]	57	M	Chemotherapy
24	2018 [26]	52	M	Resection, Radiotherapy
25	2019 [27]	52	F	Resection, Radiotherapy
26	2019 [28]	75	F	Resection, Radiotherapy
27	2020 [29]	67	M	Resection, Radiotherapy
28	2021 [30]	62	M	Biopsy, Chemotherapy, Radiotherapy
29	2024	65	M	Biopsy, Chemotherapy, Radiotherapy

**Table 2:** Differential diagnosis of pineal tumors adapted from pineal tumor workup—Medscape [31] https://emedicine.medscape.com/article/249945-workup?form=fpf#c5 (accessed on 2 June 2024).

Tumor Type		Demographics	Imaging Findings
	Germinomas	Second decade of life	Isointense on T1-weighted MRI studies, slightly hyperintense on T2, and strong homogeneous enhancement. May have evidence of calcification.
Germ cell tumors	Teratomas	Children and young adults	Heterogeneous with irregular enhancement. Includes various tissue types.
	NGGCTs	First and second decade of life	Heterogeneous. Potential presence of hemorrhage depending on specific type.
Pineal parenchymal tumors	Pineocytoma	Young adults	Hypointense to isointense on T1-weighted images, increased signal on T2 and homogeneous enhancement.
	Pineoblastoma	Typically children	Similar to pineocytomas. However, these will be irregularly shaped, large, poorly defined masses.
	Pineal parenchymal tumors	Broad spectrum, average age, 3rd decade of life	Similar to pineocytomas, but more locally invasive and heterogeneously enhancing.
	Pineal papillary tumors	Broad spectrum, average age, 4th decade of life	Mildly hyperintense on T1-weighted images. May include a cystic component.
Other lesions	Pineal metastasis	Lung, breast, GI tract	Usually concomitant with leptomeningeal metastases.
	Primary pineal melanoma	Extremely rare	Depending on the percent of melanin-containing cell: >10%—hyperintense on T1, hypointense on T2. <10%—hypointense on T1and hyperintense on T2.

## Data Availability

Data is contained within the article.
